# Wear and Friction of UHMWPE-on-PEEK OPTIMA™

**DOI:** 10.1016/j.jmbbm.2018.09.021

**Published:** 2019-01

**Authors:** Raelene M. Cowie, Adam Briscoe, John Fisher, Louise M. Jennings

**Affiliations:** a Institute of Medical and Biological Engineering, University of Leeds, Leeds LS2 9JT, UK; b Invibio Ltd. Thornton Cleveleys, Lancashire FY5 4QD, UK

**Keywords:** Arthroplasty, PEEK-OPTIMA™, UHMWPE, Wear, Friction, TKR

## Abstract

PEEK-OPTIMA™ is being considered as an alternative bearing material to cobalt chrome in the femoral component of total knee replacement to provide a metal-free implant. The aim of this study was to investigate the influence of lubricant temperature (standard rig running and elevated temperature (~36 °C)) on the wear of a UHMWPE-on-PEEK OPTIMA™ bearing couple using different lubricant protein concentrations (0%, 2%, 5%, 25% and 90% bovine serum) in a simple geometry pin-on-plate configuration. Friction was also investigated under a single temperature condition for different lubricant protein concentrations. The studies were repeated for UHMWPE-on-cobalt chrome in order to compare relationships with temperature (wear only) and lubricant protein concentration (wear and friction).

In low lubricant protein concentrations (≤ 5%) there was no influence of temperature on the wear factors of UHMWPE-on-PEEK. With 25% bovine serum, the wear factor of UHMWPE-on-PEEK reduced by half at elevated temperature. When tested in high protein concentration (90% serum), there was no influence of temperature on the wear factor of UHMWPE-on-PEEK. These temperature dependencies were not the same for UHMWPE-on-cobalt chrome.

For both material combinations, there was a trend of decreasing friction with increasing protein concentration once protein was present in the lubricant.

This study has shown the importance of the selection of appropriate test conditions when investigating the wear and friction of different materials, in order to minimise test artefacts such as polymer transfer, and protein precipitation and deposition.

## 1 Introduction

Total knee arthroplasty (TKA) is a highly successful procedure with a survivorship of > 90% at 10 years (NJR, 2016) however, up to 20% of patients are dissatisfied with their knee replacement (Bourne, 2010, Scott et al., 2010). There are many factors which could contribute to clinical success and patient satisfaction, for example, surgical positioning, implant geometry and the materials used. Recently PEEK-OPTIMA™ has been proposed as an alternative bearing material to cobalt chrome in the femoral component of total knee replacement (Cowie et al., 2016, Rankin et al., 2016, de Ruiter et al., 2017a, de Ruiter et al., 2017b). A PEEK femoral component coupled with an all-polyethylene tibial component has several potential benefits over a conventional implant. Firstly, an all-polymer knee implant would be beneficial to the ~2% of patients who exhibit metal-sensitivity reactions to their implant (Granchi et al., 2008). Further, the lower modulus of PEEK compared to cobalt chrome gives the potential to reduce implant stress shielding, which can cause bone resorption leading to failure due to loosening (Rankin et al., 2016, de Ruiter et al., 2017a, de Ruiter et al., 2017b). An all-polymer implant would be lighter weight to cobalt chrome and more similar to the weight of the natural joint. In addition, the injection moulding process used gives the potential to reduce manufacturing time and cost, which could be of particular benefit to emerging markets.

When considering any novel bearing material combination, it is important to understand the tribology, specifically, the wear and friction of the bearing materials. The response of the body to UHMWPE wear debris inducing osteolysis leading to implant loosening and ultimately failure is well understood with both the volume of the particles and their size contributing to osteolytic response (Ingham and Fisher, 2000, Fisher et al., 2001). Therefore, it is important that the volume of polymer wear debris especially in the most biologically active sub-micron size range is minimised. The friction of the bearing couple is another important consideration; in order to reduce the potential for mechanical loosening of the implant and to minimise frictional heating.

The use of poly ether-ether ketone (PEEK) either in its natural form or reinforced with carbon fibres (CFR-PEEK) has been considered as an arthroplasty bearing material for a number of applications including; finger joints (Joyce et al., 2006), intervertebral discs (Brown et al., 2010, Brown and Bao, 2012), acetabular cups (Wang et al., 1998, Wang et al., 2012, Scholes et al., 2008, Brockett et al., 2012) and tibial inserts (Kurtz and Devine, 2007, Scholes and Unsworth, 2009, Grupp et al., 2010). In these examples, PEEK has either been considered as an alternative to ultra-high molecular weight polyethylene (UHMWPE) or in the case of fingers and spine as a self-mating PEEK-on-PEEK bearing couple. In the hip, CFR-PEEK acetabular cups have been used clinically (Field, 2013) and experimental wear simulation under standard gait conditions against either metal or ceramic heads has demonstrated an improved wear performance of CFR-PEEK over UHMWPE, producing debris with a low biological response (Brockett et al., 2012, Wang et al., 2012, Scholes and Unsworth, 2009, Howling et al., 2003). In unicompartmental knee replacements, low wear rates have been measured experimentally in highly conforming implants (Scholes and Unsworth, 2009). However, there has been concern expressed regarding the use of CFR-PEEK in high contact stress situations for example, in the knee, when an UHMWPE tibial insert is replaced with a CFR-PEEK tibial insert and the implant has either a low conformity or is mal-positioned. The high contact stresses produced give potential for gross failure of the material (Grupp et al., 2010, Evans et al., 2014, Brockett et al., 2016, Brockett et al., 2017). Experimental wear simulation of UHMWPE-on-PEEK-OPTIMA™ where the PEEK is intended to be used as an alternative to cobalt chrome for the femoral component of a total knee replacement has shown UHMWPE wear rates equivalent to UHMWPE-on-cobalt chrome (Cowie et al., 2016, East et al., 2015, Baykal et al., 2016).

When considering the tribology of novel bearing material combinations, it is evident that a multitude of factors including cross shear, contact pressure (Brockett et al., 2016, Kang et al., 2008) lubricant (Scholes and Joyce, 2013), surface topography (Lancaster et al., 1997) and environmental conditions including the temperature of the test (Liao et al., 2003) can influence the tribology of arthroplasty bearing materials and that the influence of these variables may differ depending on the material combination. Different experimental approaches have been taken to investigate how these variables influence tribology. Simple geometry configurations such as pin-on-plate or pin-on-disc tribometers provide screening devices which allow different materials to be tested and the influence of variables to be systematically investigated. The flat-on-flat configuration with simple loading and motion profiles means that the interactions of materials can be determined without the influence of component geometry or setup (Kang et al., 2008, Galvin et al., 2006, ASTMF732, 2017).

The aim of this study was to investigate the wear and friction of an UHMWPE-on-PEEK bearing couple using a series of pin-on-plate studies. Specifically, to investigate the influence of lubricant temperature (standard rig running and elevated temperature) and different lubricant protein concentrations on the wear of UHMWPE-on-PEEK. A secondary aim was to investigate the friction of UHMWPE-on-PEEK under a single temperature condition (standard rig running temperature) for different lubricant protein concentrations. The studies were repeated for UHMWPE-on-cobalt chrome in order to compare relationships with temperature (wear only) and lubricant protein concentration (wear and friction).

## 2 Materials

The pins used were GUR 1020 UHMWPE (conventional) which was machined into a truncated cone geometry with an 8 mm flat contact face. The plates were either highly polished cobalt chrome (initial mean surface roughness, Ra <0.01 µm) or injection moulded, implant grade, unfilled (natural) PEEK-OPTIMA™ (Invibio Ltd, UK) (Ra ~ 0.04 µm). Prior to the start of the study, the polymeric materials were soaked in sterile water to maximise their moisture uptake. The pins were soaked for a minimum of 2 weeks (Yao et al., 2003) and the plates for minimum 12 weeks (Wang et al., 2012). The lubricant used was new born calf serum which was diluted to a final concentration using sodium azide solution to minimise bacterial degradation.

## 3 Methods

### 3.1 Pin-on-plate wear tests

**Fig. 1:**
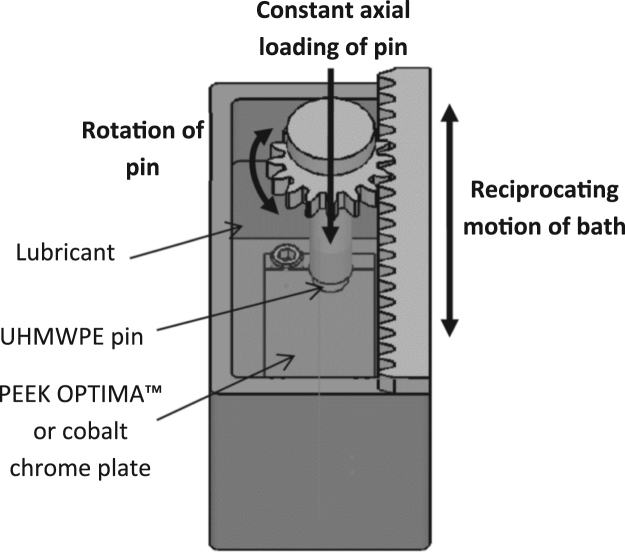
Schematic of a bath in the pin on plate rig.

Experimental wear simulation was carried out using a 6-station multi-axial pin-on-plate reciprocating rig (Fig. 1) (Galvin et al., 2006). The cobalt chrome or PEEK-OPTIMA™ (PEEK) plate was held in a lubricant containing bath which was reciprocated at 1 Hz over a stroke length of 20 mm. The UHMWPE pin was clamped into a pin holder through which a constant axial load of 160 N was applied via a mass carrying cantilever mechanism. To create multi-directional motion, as the bath reciprocated, the pin rotated via a rack and pinion mechanism (± 20°). The kinematic conditions were consistent for all the wear studies and were chosen to reflect the average contact pressure (3.18 MPa) and cross shear (0.039) in total knee replacements (Fisher et al., 2004). Bovine serum was diluted to concentrations of 2 (1.2 g/l), 5 (3 g/l), 25 (15 g/l) and 90% (54 g/l) using sodium azide solution to reach a final concentration of sodium azide of 0.03% (v/v). For the 0% study, the test was carried out in sterile water. To investigate the influence of lubricant temperature, studies were carried out at either room temperature with no intervention (standard rig running temperature) as per standard practice at Leeds (McEwen et al., 2005) or at elevated temperature (~36 °C for soak control) as per the ISO standard for wear testing of knee prostheses (ISO14243-1:2014) and ASTM F732 for wear testing of polymeric materials used in total joint prostheses (ASTMF732, 2017). The elevated temperature was achieved by incorporation of an enclosure heater system into the rig which raised the temperature of the environment. The heater system comprised two enclosure heaters (Cirrus 25 heater, DBK, Germany) incorporating both a heating element and a fan in a single unit to aid even distribution of the heat around the rig. The feedback system used a CAL 9900 PID temperature controller (West Control Solutions, IL, USA), the input to which came from a K-type thermocouple placed in the soak control. The temperature controller turned the heaters on or off to maintain the temperature of the soak control at a desired set-point.

The matrix of test conditions investigated is shown in Table 1. For the test carried out at standard rig running temperature in 25% serum, one sample was damaged during the wear simulation so only 5 repeats were carried out. Once the data had been reviewed, it was deemed unnecessary to carry out the test in 2% serum at elevated temperature or to carry out further repeats in 0% serum as for the tests in 0% serum as the variability in the results was low and followed a similar trend.

**Table 1:** The test matrix showing the lubricant protein concentrations and temperatures studied and the number of samples (N) investigated in the wear simulation.

		Plate material	Lubricant protein concentration (%)				
			0	2	5	25	90
Temperature	Standard rig running	PEEK-OPTIMA™	N=6	N=6	N=6	N=6	N=6
		Cobalt chrome	N=6	N=6	N=6	N=5	N=6
	Elevated	PEEK-OPTIMA™	N=3		N=6	N=6	N=6
		Cobalt chrome	N=3		N=6	N=6	N=6

All the wear tests were carried out for 1 million cycles with the wear of the UHMWPE pins assessed by gravimetric analysis every 0.3 MC. Prior to weighing, the pins were cleaned in 70% propan-2-ol in an ultrasonic bath before being allowed to stabilise for a minimum of 48 h in a temperature and humidity controlled environment (20 ± 1 °C, 40 ± 5%). The same cleaning and weighing protocol was used at each measurement point. Measurements were carried out using a Mettler Toledo AT21 high-precision (0.001 mg resolution) digital microbalance (Mettler Inc., OH, USA) using 2 unloaded soak controls maintained in the same lubricant and environment as that used in the wear test to compensate for the uptake of moisture. Measurements were taken until 5 consecutive measurements fell within a range of ± 5 µg. The change in weight of the UHMWPE pins was converted to a volume loss using a density of 0.934 g/cm^3^ for GUR 1020 UHMWPE. The wear factor, *k*, of the pins was calculated using the following equation (Galvin et al., 2006):

$$k=\frac{V}{\mathrm{PX}}$$

Where *k* is the wear factor (mm^3^/Nm), *V* is the volumetric wear (mm^3^), *P* is the applied load (N) and *X* the sliding distance (m). The wear of the PEEK-OPTIMA™ plates was also assessed using the same cleaning and weighing protocol however, due to inconsistencies in the uptake of moisture by the PEEK, the measurements proved to be unreliable and hence have not been reported.

The surface topography of the plates was assessed using a PGI 800 contacting Form Talysurf (Taylor Hobson, Leicester, UK) with a 2 µm conical tip stylus. Five traces were taken perpendicular to the direction of the wear test, Least Squares Line form removal was used with using Gaussian filtering and a 0.25 mm upper cutoff in line with ISO 4288:1998. The mean surface roughness (Ra) of the plates was assessed prior to and post-test.

The temperature of the soak control and the bulk lubricant temperature in the baths was measured daily using a digital thermometer (Fluke 51 II, WA, USA).

Mean ± 95% confidence limits were determined for wear factor, bulk lubricant temperature and the mean surface roughness (Ra) of the plates. In order to determine the influence of an elevated lubricant temperature on the wear of UHWMPE-on-PEEK at different protein concentrations, the wear factor data was statistically analysed and compared for each protein concentration at standard rig running temperature versus elevated temperature using a one way ANOVA (p < 0.05). This was repeated for UHMWPE-on-cobalt chrome.

### 3.2 Pin-on-plate friction tests

**Fig. 2:**
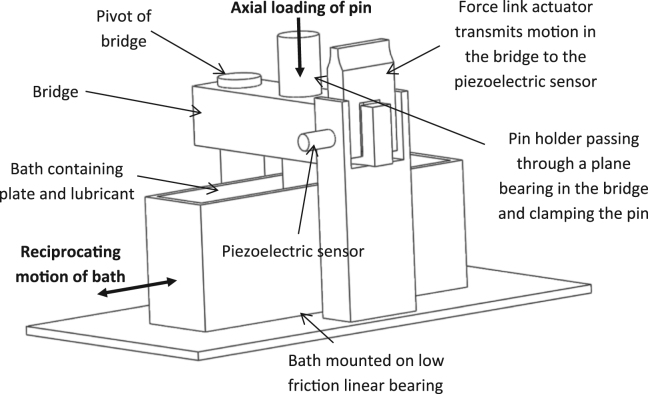
A schematic of the pin on plate friction rig.

The friction between the different materials at different protein concentration levels was determined using a uniaxial pin-on-plate friction rig (Fig. 2) similar to that previously described by Forster and Fisher (1999). The PEEK-OPTIMA™ or cobalt chrome plates were held in a bath containing lubricant mounted on a low friction linear bearing. The bath was continuously driven by a motor and reciprocated over a stroke length of 20 mm at 0.5 Hz. The UHMWPE pin was held in a holder and an axial load of 160 N was applied through the pin to reflect the contact pressure used in the wear simulation. The pin holder passed through a plain bearing in a bridge. One end of the bridge could pivot via a low friction bearing, the other was free to move. As the bath reciprocated, movement of the free end of the bridge was transmitted to a piezoelectric force sensor via a force link actuator, the output voltage from which was collected using LabView (National Instruments, TX, USA) and converted to a frictional force (*F*_*R*_) using previously determined calibration factors which took into account friction in the system. To calculate the coefficient of friction, µ, the following equation was used:

$$µ=\frac{F_{R}}{F_{N}}$$

Where µ is the coefficient of friction, *F*_*R*_ is the frictional force (N) and *F*_*N*_ is the normal reaction force to the applied load (N).

All tests were carried out at room temperature due to a limitation of the rig using bovine serum diluted to 0%, 2%, 5%, 25% and 90%. The dynamic friction was assessed once the system had reached a steady state (5 min). At each test condition, each set of bearing couples was assessed three times and six bearing couples were tested for each material combination.

The mean ± 95% confidence limits were determined for the coefficient of friction at each lubricant protein concentration.

The data associated with this study is openly available through the University of Leeds Data Repository (Cowie and Jennings, 2018).

## 4 Results

### 4.1 Experimental wear simulation

**Fig. 3:**
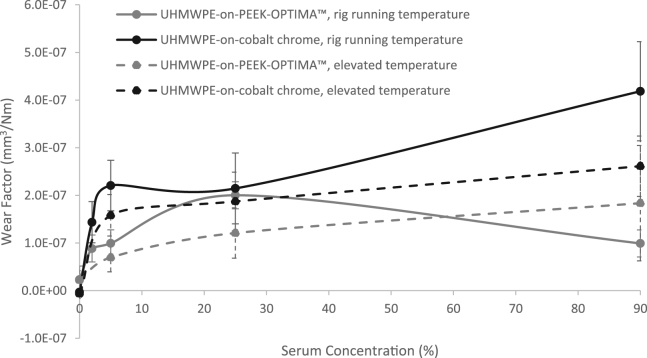
Mean Wear Factor (mm^3^/N m) ± 95% confidence limits of UHMWPE-on-PEEK-OPTIMA™ and UHMWPE-on-cobalt chrome bearing couples at standard rig running and elevated temperatures and at different serum concentrations.

The mean wear factors of the UHMWPE-on-PEEK and UHMWPE-on-cobalt chrome bearing couples under all test conditions are shown in Fig. 3.

Without protein present in the lubricant (0% serum), polymer transfer was evident on the cobalt chrome plates and the wear factors were very low for both material combinations irrespective of lubricant temperature (p > 0.79 UHMWPE-on-PEEK, p > 0.34 UHMWPE-on-cobalt chrome). The addition of serum to the lubricant even at very low concentrations (2%) increased the wear factor of the UHMWPE-on-PEEK and UHMWPE-on-cobalt chrome bearing couples. Polymer transfer was however, still visible and was more apparent on the cobalt chrome plates tested in 5% serum at both room and elevated temperature than the PEEK plates. In 5% serum the wear factor of UHMWPE-on-PEEK was not significantly different at standard rig running or elevated temperature conditions (p > 0.18). However, the wear factor of UHMWPE-on-cobalt chrome was significantly lower at the elevated temperature (p < 0.04) compared to standard rig running temperature.

After 1 MC wear simulation in 25% serum at the standard rig running temperature, the wear factor of the UHMWPE-on-PEEK was 2.00 × 10^−7^ ± 1.08 × 10^−7^ mm^3^/N m, and the wear factor of the UHMWPE-on-cobalt chrome bearing couple was 2.15 × 10^−7^ ± 7.43 × 10^−8^ mm^3^/N m. In 25% serum increasing the temperature of the test environment approximately halved the wear factor of the UHMWPE-on-PEEK bearing couple (9.93 ×10^−8^ ± 2.96 ×10^−8^ mm^3^/N m, p < 0.04), whereas increasing lubricant temperature had no influence on the wear factor of the UHMWPE-on-cobalt chrome bearing couple (1.87 ×10^−7^ ± 6.14 ×10^−8^ mm^3^/N m, p > 0.46).

Similar to the lower protein concentration of 5%, testing in 90% serum at different temperatures did not significantly influence the wear factor of UHMWPE-on-PEEK (p > 0.25) and the wear factor of UHMWPE-on-cobalt chrome was significantly lower at the elevated temperature (p < 0.01) compared to standard rig running temperature. In 90% serum at standard rig running temperature, a deposit, thought to be protein, was evident in the wear area of the cobalt chrome plates and the UHMWPE pins had evidence of adhesive wear and detachment/reattachment of UHMWPE to their surface. In 90% serum at elevated temperature, an additional unstable layer of protein was visible outside of the wear area on the cobalt chrome plates. On the surface of the PEEK, a wear scar was visible but there was no discernible protein deposition or precipitation. However, for both bearing couples in high serum concentration at elevated temperature, a precipitate of protein was visible in the lubricant which appeared cloudy.

Under elevated temperature conditions, increasing protein concentration led to a trend of increasing wear factor, this trend was the same for both the UHMWPE-on-PEEK and UHMWPE-on-cobalt chrome bearing couples; under standard rig running temperature conditions, the trend for the wear of UHMWPE-on-PEEK was not the same as that for UHMWPE-on-cobalt chrome bearing couples, with a reduction in wear factor at high serum concentration (Fig. 3).

Images of the wear scars on the PEEK plates and the deposits on the CoCr plates are available through the University of Leeds data repository (Cowie and Jennings, 2018).

After 1 MC wear simulation, there was linear scratching visible on all the PEEK plates and burnishing caused by the pin under all test conditions whereas, the cobalt chrome plates had discrete scratches on the surface. The pre- and post-test mean surface roughness of the PEEK and cobalt chrome plates are shown in Table 2. In the tests carried out in water, machining marks on the contact surface of the pins were still visible at the conclusion of the test; in all other studies, after 1MC, the pins had a polished region where they had contacted the plate.

**Table 2:** Pre- and post-test mean surface roughness (Ra) with 95% confidence limits of PEEK Optima™ and cobalt chrome plates.

Parameters	Pre-test	0%		2%	5%		25%		90%	
		Room	Elevated	Room	Room	Elevated	Room	Elevated	Room	Elevated
PEEK-OPTIMA™	0.035	0.174 ± 0.033	0.244 ± 0.144	0.039 ± 0.010	0.220 ± 0.261	0.106 ± 0.075	0.196 ± 0.120	0.148 ± 0.081	0.266 ± 0.153	0.270 ± 0.181
Cobalt Chrome	0.006	0.008 ± 0.004	0.008 ± 0.001	0.007 ± 0.005	0.026 ± 0.015	0.036 ± 0.046	0.010 ± 0.005	0.019 ± 0.020	0.015 ± 0.010	0.017 ± 0.006

The bulk lubricant temperature of the standard rig running temperature tests is detailed in Table 3. For both bearing couples, there was a trend of decreasing lubricant temperature with increasing protein concentration. The bulk lubricant temperature for the elevated temperature study has been added as supplementary data (Cowie and Jennings, 2018) as it is believed that fluctuations in the measured lubricant temperature may be caused by local variations in the heater system rather than as a result of changes to the test components or environment.

**Table 3:** Mean bulk lubricant temperature (°C) with 95% confidence limits for standard rig running temperature tests.

Plate material	Lubricant protein concentration (%)				
	0	2	5	25	90
PEEK- OPTIMA™	29.1 ± 2.8	28.3 ± 0.6	27.2 ± 0.9	27.5 ± 1.0	26.6 ± 1.1
Cobalt Chrome	28.5 ± 3.5	27.8 ± 1.2	26.6 ± 0.7	26.7 ± 1.4	26.0 ± 1.4

### 4.2 Friction study

**Fig. 4:**
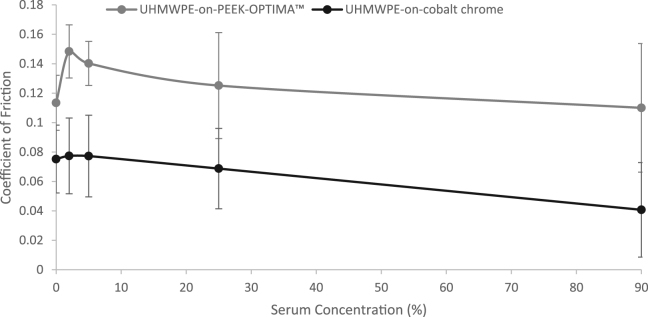
Mean coefficient of friction ± 95% confidence limits of UHMWPE-on-PEEK-OPTIMA™ and UHMWPE-on-cobalt chrome bearing couples, under different serum concentrations.

The mean coefficient of friction of the UHMWPE-on-PEEK and UHMWPE-on-cobalt chrome bearing couples with respect to increasing protein concentration is shown in Fig. 4. With increasing protein concentration (above 2%), there was a trend for decreasing friction for both material combinations. In 25% serum, the coefficient of friction was 0.13 ± 0.04 for UHMWPE-on-PEEK and 0.07 ± 0.03 for UHMWPE-on-cobalt chrome bearing couples.

## 5 Discussion

To investigate the influence of temperature on the wear of UHMWPE against PEEK and cobalt chrome using different lubricant protein concentrations, pin-on-plate studies were carried out at both standard rig running (room) temperature and elevated temperature (~36 °C).

Temperature had no influence on the wear of either UHMWPE-on-PEEK or UHMWPE-on-cobalt chrome when a lubricant with no protein (i.e. water) was used. The wear factors of both material combinations were low, at both standard rig running and elevated temperature, consistent with previous studies of metal-on-UHMWPE (Tateiwa et al., 2006, Good et al., 2000). Previous studies have shown water lubricated systems to produce large flakes of UHMWPE wear debris, not the clinically relevant sub-micron wear particles (Besong et al., 1999, Wang et al., 1996).

As little as 2% serum increased the wear factors for both material combinations tested at both standard rig running temperature and elevated temperature. In 5% serum, there was no influence of temperature on the wear factor of UHMWPE-on-PEEK. This was not the case for UHMWPE-on-cobalt chrome, for which the wear factor was significantly lower at elevated temperature compared to standard rig running temperature. The reason for this was unclear, but could be related to the extent of polymer transfer combined with effects of protein precipitation and deposition. Polymer transfer due to adhesive wear was visible on the surface of the cobalt chrome plates at 2% and 5% serum regardless of temperature, suggesting insufficient boundary lubrication (Wang et al., 1996, Cooper et al., 1993).

The most clinically relevant protein lubricant concentration used in this study was 25% serum (Wang et al., 2004, Galvin et al., 2007). After 1MC of wear simulation in 25% serum at standard rig running temperature, the wear factor of the UHMWPE-on-cobalt chrome bearing couple was consistent with previous studies of UHMWPE-on-metal carried out under similar environmental conditions (Kang et al., 2008). With 25% serum, the wear factor of UHMWPE-on-PEEK reduced by half at elevated temperature. This lower wear factor in the UHMWPE-on-PEEK bearing couple at elevated temperature was possibly a result of protein precipitation and deposition on the articulating surfaces. The resulting deposition may have created a protein rich layer artificially protecting the surfaces against wear. In contrast, there was no influence of temperature on the wear factor of UHMWPE-on-cobalt chrome at this concentration. Although the lubricant temperature has not been presented for the elevated temperature study, it was possible that due to the higher friction of the all-polymer bearing couple, the bulk lubricant temperature was higher in the UHMWPE-on-PEEK study. This elevated bulk lubricant temperature may have accelerated protein deposition in the UHMWPE-on-PEEK bearing couple (Liao et al., 2003; Lu and McKellop, 1997).

When tested in high protein concentration lubricant (90% serum), there was no significant influence of temperature on the wear factor of the UHMWPE-on-PEEK. However, the wear factor of UHMWPE-on-cobalt chrome was significantly lower at elevated temperature and an additional unstable layer of protein was visible outside of the wear area on the cobalt chrome plates, as well as a deposit understood to be protein in the wear area and a precipitate of protein in the bath. High protein concentrations have been associated with increased protein precipitation, which may reduce the boundary lubricating properties of the serum if the precipitated protein were to form a compacted solid that becomes trapped between the articulating surfaces (Wang et al., 2004, Liao et al., 1999). However, the volume of lubricant also has a role in the precipitation rate and the influence of protein precipitation on wear; the decrease in wear factor of UHMWPE-on-cobalt chrome suggests that in this study, the lubricant volume was sufficiently high to maintain the concentration of protein in the lubricant (Wang et al., 2004). At elevated temperature, there is potential for degradation of the protein rich lubricant and for protein to come out of solution, forming a precipitate which may adhere to the articulating surfaces. There is potential for this precipitate to artificially protect the articulating surfaces changing the lubrication regime of the bearing couple resulting in a lower wear of UHMWPE (Lu and McKellop, 1997). Changing the test temperature and lubricant protein concentration had different effects on wear factor for the two material combinations of interest which suggest that the protein precipitation rate and resulting effects are material dependant.

For UHMWPE-on-cobalt chrome at both standard rig running and elevated temperatures, there was a trend of increasing wear factor with increasing protein concentration. These findings corroborate with those of several previous pin-on-plate (Galvin, 2003) and whole joint wear simulation studies (Good et al., 2000, Hyde et al., 2016) run at room temperature however, other studies also carried out at room temperature have reported an inverse relationship between protein concentration and wear (Liao et al., 1999, Liao et al., 2003, Muratoglu et al., 2004). Differences in simulation systems and test protocols, and the use of additives such as EDTA and antibiotics to the lubricant may have contributed to the different test outcomes.

At elevated temperature, the wear of both UHMWPE-on-PEEK and UHMWPE-on-cobalt chrome bearing couples showed a trend of increasing wear with increasing protein lubricant concentration. This was similar to the findings of Tatiewa et al. in a metal-on-UHMWPE hip simulation study (Tateiwa et al., 2006). This may have been due to a converse effect of precipitation - excessive precipitation depleting the soluble proteins to such an extent that there was insufficient boundary lubrication, hence artificially accelerating the adhesive wear (Lu and McKellop, 1997).

The continuous running of experimental wear simulators can also contribute to lubricant heating which can in turn cause degradation of the protein rich bovine serum lubricant leading to test artefacts (Liao et al., 2003). Experimentally, different bearing material combinations have been shown to have different influences on bulk lubricant temperature due to frictional heating (Cowie et al., 2016) and, in patients with joint replacements, the intra-articular temperature can vary depending on the bearing materials used (Pritchett, 2011). The ISO standards for wear testing of knee prostheses (ISO14243-1, 2014, ISO14243-1, 2014) suggest running experimental wear simulation at 37 ± 2 °C to reflect core body temperature. At elevated test temperatures however, there is potential for test artefacts due to heating and subsequent degradation of protein rich lubricant, this effect is emphasised by continuous running of simulators.

Under all the wear test conditions, the PEEK plates became scratched but, over the relatively short duration of testing, there was no apparent influence on wear rate which remained linear. Wear is dependant on surface topography; previous studies of UHMWPE-on-metal have demonstrated an exponential relationship between wear factor and either Ra or Rp and have shown the scratch orientation and geometry, specifically the lip height of the scratches to influence wear (Lancaster et al., 1997, Minakawa et al., 1998). The pre-test mean surface roughness (Ra) of the plates was 0.035 and 0.006 µm for PEEK and cobalt chrome respectively, although the roughness of the two materials differed, the magnitude of the roughness of the PEEK plates was below that which would influence wear factor (Lancaster et al., 1997). Following the wear test, the direction of the resultant scratching on the PEEK plates parallel to the direction of the wear test is consistent with knee simulation studies of a PEEK OPTIMA™-on-UHMWPE implant. In this knee simulator study, the scratching of the PEEK implant had no influence on wear rate (Cowie et al., 2016). Further studies will be necessary to fully describe the relationship between surface topography and wear factor for UHMWPE-on-PEEK and longer duration studies will be necessary to confirm whether the surface of the PEEK will deteriorate further or whether the scratches on the surfaces will influence wear rate in the longer term.

For both material combinations, there was a trend of decreasing friction with increasing protein concentration once protein was present in the lubricant. Brockett et al. showed the inverse trend in both metal-on-UHMWPE in a pendulum friction simulator (Brockett et al., 2007) and metal or ceramic-on-CFR PEEK hip replacements (Brockett et al., 2012). However, Yao et al. reported a similar trend with a decrease in coefficient of friction between 25% and 100% serum in a pin-on-disc study (Yao et al., 2003). Hence these differences may be explained by the differing simulation methods.

There were several limitations associated with this study; firstly, the tests were carried out in a simple geometry configuration. Pin-on-plate tests are invaluable for screening materials and allow a single variable to be systematically investigated. In this study the variables of lubricant temperature and protein concentration have been systematically investigated for 2 bearing material combinations. However, in a joint replacement, the complex geometry and loading profiles will have a role in the tribology of the implant. In this study, it was only possible to assess the wear of the UHMWPE pins. Attempts were made to assess the wear of the PEEK however, inconsistences in moisture uptake of the PEEK as previously reported by Brockett et al. (2012) coupled with very low wear meant that both geometric and gravimetric assessment techniques proved unreliable. The approach used for heating the lubricant involved raising the temperature of the test environment. The majority of commercially available joint simulators heat test cells individually to achieve an elevated lubricant temperature and to ensure the temperature in each test cell is consistent. The input temperature was the same for both material combinations. Further, the friction study could only be reliably carried out at room temperature and the tests were relatively short-term (10 min) therefore, as the wear and friction studies were carried out independently, it is not known whether the polymer transfer, protein deposition or protein precipitation had an influence on the coefficient of friction (Scholes and Unsworth, 2006).

## 6 Conclusion

The influence on wear of lubricant temperature at different protein concentrations has been systematically investigated for UHMWPE-on-PEEK, and for UHWMPE-on-cobalt chrome bearing couples. The resulting wear relationships were complex, and different for the two material combinations, showing the importance of systematic investigations to fully understand fundamental tribological relationships of different material combinations. This study has shown the importance of the selection of appropriate test conditions when investigating the wear and friction of different materials, in order to minimise test artefacts.

## Funding

This work was supported by Invibio Knees Ltd and the Innovation and Knowledge Centre in Regenerative Therapies and Devices funded by the EPSRC, TSB and BBSRC (grant number EP/J017620/1).﻿ It was partially funded through WELMEC, a centre of Excellence in Medical Engineering funded by the Wellcome Trust and EPSRC (grant number WT 088908/Z/09/Z) a﻿nd supported by the EPSRC Centre for Innovative Manufacturing in Medical Devices (grant number EP/K029592/1)﻿. The research is supported by the National Institute for Health Research (NIHR) Leeds Musculoskeletal Biomedical Research Unit. The views expressed are those of the author(s) and not necessarily those of the NHS, the NIHR or the Department of Health. The PEEK-OPTIMA™ plates were provided by Invibio Knees Ltd. Thanks to Phil Wood and his team for technical assistance.

## Competing interests

AB is a paid employee of Invibio Ltd., JF is a consultant to Invibio Ltd.
